# Effect of Mukitake mushroom (*Panellus serotinus*) on the pathogenesis of lipid abnormalities in obese, diabetic *ob/ob* mice

**DOI:** 10.1186/1476-511X-12-18

**Published:** 2013-02-14

**Authors:** Nao Inoue, Masashi Inafuku, Bungo Shirouchi, Koji Nagao, Teruyoshi Yanagita

**Affiliations:** 1 Department of Applied Biochemistry and Food Science, Saga University, Saga 840-8502, Japan; 2 Department of Food Function and Health, Tohoku University, Miyagi, 981-8555, Japan; 3 Department of Mangroves and Bio-resources, University of the Ryukyus, Okinawa, 903-0213, Japan; 4 Department of Bioscience and Biotechnology, Kyushu University, Fukuoka, 812-8581, Japan; 5 Department of Health and Nutrition Sciences, Nishikyushu University, Kanzaki 842-8585, Japan

**Keywords:** *Panellus serotinus*, Atherogenic index, Nonalcoholic fatty acid disease, *ob/ob* mice

## Abstract

**Background:**

Various mushrooms have been used in folk medicine for the treatment of lifestyle diseases in eastern countries, and several compounds that modulate the immune system, lower blood lipid levels, and inhibit tumor and viral action have been isolated. The fruiting body of *Panellus serotinus* (Mukitake) is recognized in Japan as one of the most delicious edible mushrooms, and previous studies have demonstrated that the dietary intake of powdered whole Mukitake or Mukitake extracts prevents the development of non-alcoholic fatty liver disease (NAFLD) in leptin-resistant *db/db* mice. In the present study, we evaluated the effect of the Mukitake diet on the pathogenesis of metabolic disorders in leptin-deficient *ob/ob* mice.

**Results:**

After 4 weeks of feeding, hepatomegaly, hepatic lipid accumulation, and elevated hepatic injury markers in the serum were markedly alleviated in Mukitake-fed *ob/ob* mice compared with control mice. Moreover, the mild hyperlipidemia in control *ob/ob* mice was attenuated and the elevated atherogenic index was reduced in Mukitake-fed *ob/ob* mice. These effects were partly attributable to the suppression of hepatic lipogenic enzyme activity due to the Mukitake diet.

**Conclusion:**

The current results showed that Mukitake supplementation is beneficial for the alleviation of NAFLD and dyslipidemia in obese, diabetic *ob/ob* mice.

## Background

Diet contributes to the development and prevention of lifestyle-related diseases, and various mushrooms have been used in folk medicine for the treatment of lifestyle-related diseases in eastern countries [1-3]. Several lines of evidence support the nutraceutical effect of edible mushrooms, and many compounds that modulate the immune system, lower blood lipid levels, and inhibit tumors and viral action have been isolated from various mushrooms, such as Shiitake and Hatakeshimeji [1-6]. *Panellus serotinus* belongs to the same family of mycelia as *Lentinus edodes* (Shiitake) and *Lyophyllum decastes* (Hatakeshimeji), and its fruiting body (Mukitake) is recognized in Japan as one of the most delicious edible mushrooms. The technology for the artificial cultivation of Mukitake in plastic greenhouses has recently been developed [7] and has enabled the constant provision of Mukitake mushrooms in the market.

Metabolic syndrome, which comprises a cluster of metabolic abnormalities, such as hyperlipidemia, diabetes mellitus, and hypertension, is a widespread and increasingly prevalent disease in industrialized countries and contributes to the increase in cardiovascular morbidity and mortality [8,9]. Nonalcoholic fatty liver disease (NAFLD) is often associated with features of metabolic syndrome and is emerging as the most common liver disease worldwide [10-13]. Dyslipidemia is a lipid abnormality in the blood, and hyperlipidemia is prevalent due to westernized diets and the disadvantages of modern lifestyles. Insulin resistance can also lead to dyslipidemia. Many studies evaluating the effects of functional food components on the pathogenesis of obesity-related metabolic disorders have been carried out in genetic leptin-resistant *db/db* mice and leptin-deficient *ob/ob* mice [14,15]. *db/db* mice have a missense mutation in the leptin receptor gene [14], and *ob/ob* mice have an inherited deficiency of the leptin gene [15], and they suffer from hyperphagia and develop a syndrome with multiple metabolic and hormonal disorders, including NAFLD and dyslipidemia, which shares many features with human metabolic syndrome. A recent study suggested that pathological features of liver lesions and the response to diets are slightly different between *db/db* and *ob/ob* mice [16].

In previous studies, we demonstrated that the dietary intake of powdered whole Mukitake or Mukitake extracts prevented the development of NAFLD, partly through the suppression of hepatic lipogenesis and normalized adipocytokine profiles, in leptin-resistant *db/db* mice [17,18]. In the present study, we evaluated the effect of the Mukitake diet on the pathogenesis of metabolic disorders in leptin-deficient *ob/ob* mice.

## Materials and methods

### Animals and diets

All aspects of the experiment were conducted according to the guidelines provided by the ethical committee for experimental animal care at Saga University. Five-week-old male C57BL/6J mice and *ob/ob* mice were purchased from Japan SLC (Shizuoka, Japan). The mice were individually housed in plastic cages in a temperature-controlled room (24°C) under a 12-h light/dark cycle. The basal semisynthetic diets were prepared according to recommendations of the AIN-76 [19] (Table 1). Mukitake mushrooms were provided by the Forestry Research Institute of the Saga Prefecture, and general components of the samples were routinely determined according to official AOAC methods. The *ob/ob* mice were assigned into two groups (six mice each) that were fed one of two diets (Table 1): a semisynthetic AIN-76 diet (Control group) or a semisynthetic AIN-76 diet supplemented with 10% air-dried Mukitake powder instead of casein, corn starch, and cellulose (Mukitake group). C57BL/6J mice (n = 6), the progenitors of the *ob/ob* mice, were fed the same diet as the *ob/ob* mice in the Control group (Normal group). The mice received the diets ad libitum using Rodent CAFE (KBT Oriental Co. Ltd., Saga, Japan) for 4 weeks. At the end of the feeding period, the mice were sacrificed by exsanguination from the heart under pentobarbital sodium salt anesthesia after a 9-h starvation period. Abdominal white adipose tissue and livers were excised immediately, and the serum was separated from the blood.

**Table 1:** Composition of experimental diets

**Ingredients**	**Control**	**Mukitake**
	(%)	
Casein	20.0	18.8
Corn starch	15.0	8.9
Cellulose	5.0	3.1
Mineral mixture (AIN 76)	3.5	3.5
Vitamin mixture (AIN 76)	1.0	1.0
DL-Methionine	0.3	0.3
Choline bitartrate	0.2	0.2
Corn oil	5.0	5.0
Mukitake powder *	0.0	10.0
Sucrose	50.0	49.2

* Carbohydrate: 62.5%, Fiber: 19.2%, Protein: 12.5%, Ash: 3.8%, Fat: 1.9%.

### Measurement of serum parameters

Serum lipoproteins were analyzed using an on-line dual enzymatic method for the simultaneous quantification of triglyceride and cholesterol using high-performance liquid chromatography at Skylight Biotech Inc. (Akita Japan), as described elsewhere [20]. The activities of aspartate aminotransferase (AST) and alanine aminotransferase (ALT) in the serum were measured using commercial enzyme assay kits (Wako Pure Chemicals, Tokyo, Japan). Serum adiponectin and insulin levels were measured using commercial mouse ELISA kits (Otsuka Pharmaceutical Co. Ltd., Tokyo, Japan; Shibayagi Co. Ltd., Gunma, Japan, respectively).

### Measurement of triglyceride and cholesterol levels in the liver

Liver lipids were extracted, and the concentrations of triglyceride and cholesterol were measured as described previously [21,22].

### Assays of hepatic enzyme activity

The preparation of hepatic subcellular fractions was described previously [23]. The enzyme activities of fatty acid synthase (FAS), glucose 6-phosphate dehydrogenase (G6PDH), and malic enzyme in the cytosomal fraction, carnitine palmitoyltransferase (CPT) in the mitochondrial fraction, and phosphatidate phosphohydrolase (PAP) in the microsomal fraction were determined as described elsewhere [24].

### Statistical analysis

All values are expressed as the means ± standard error. The data were analyzed using a one-way ANOVA, and all differences were analyzed using the Tukey-Kramer post-hoc test (KaleidaGraph; Synergy Software, Reading, PA, USA). Differences were considered to be significant at *p* < 0.05.

## Results and discussion

### Effect of dietary Mukitake on the pathogenesis of NAFLD in *ob/ob* mice

**Figure 1:**
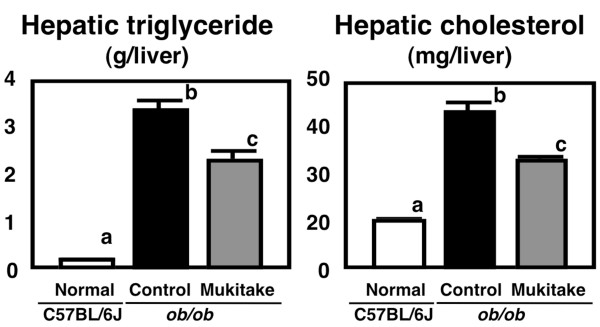
**Hepatic lipid levels in C57BL/6J and *ob*/*ob *mice.** Mice were fed the control diet or Mukitake diet for 4 weeks. Values are expressed as the mean ± standard error for six mice. See Table 1 for the composition of diets. ^abc^ Different superscripted letters indicate a significant difference at *P* < 0.05.

**Figure 2:**
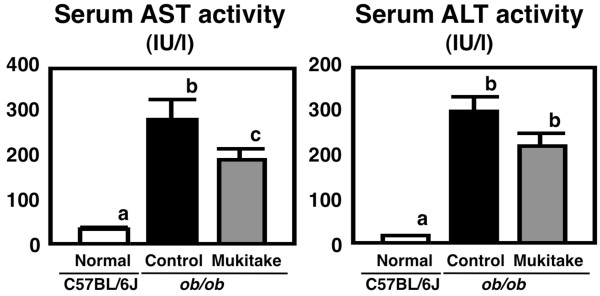
**Hepatic injury marker activities in C57BL/6J and *ob*/*ob *mice.** Mice were fed the control diet or Mukitake diet for 4 weeks. Values are expressed as the mean ± standard error for six mice. See Table 1 for the composition of diets. ^abc^ Different superscripted letters indicate a significant difference at *P* < 0.05.

The food intake (Normal, 76.8 ± 2.8; Control, 129 ± 5 g, *p* < 0.05) and final body weight (Normal, 24.6 ± 0.5; Control, 41.2 ± 0.7 g, *p* < 0.05) were significantly higher in the control diet-fed *ob/ob* mice than the C57BL/6J mice after the 4-week feeding period. After being fed the control diets for 4 weeks, the *ob/ob* mice developed marked abdominal obesity (Normal, 5.97 ± 0.22; Control, 22.3 ± 0.5 g/100 g bw, *p* < 0.05) and hepatomegaly (Normal, 3.78 ± 0.09; Control, 10.7 ± 0.3 g/100 g bw, *p* < 0.05). Although the two groups of *ob/ob* mice did not differ in initial body weight (Control, 35.4 ± 1.1; Mukitake, 35.5 ± 0.8 g) or food intake (Control, 129 ± 5; Mukitake, 129 ± 3 g), the liver weight was 21% lower in Mukitake-fed *ob/ob* mice (Control, 10.7 ± 0.3; Mukitake, 8.42 ± 0.18 g/100 g bw, *p* < 0.05). The reduction was associated with decreased triglyceride (32%) and cholesterol (31%) accumulation in the liver (Figure 1). The effects of dietary Mukitake on hepatic injury marker levels were also evaluated, and the activity of ALT tended to decrease (by 26%) and AST activity was markedly decreased (by 32%, *p* < 0.05) in the serum of Mukitake-fed *ob/ob* mice compared to control-fed *ob/ob* mice (Figure 2). Consistent with previous reports that indicated the alleviation of hepatomegaly and hepatic steatosis in Mukitake-fed *db/db* mice [17,18], dietary Mukitake also protected *ob/ob* mice from the development of NAFLD.

### Effect of dietary Mukitake on the pathogenesis of dyslipidemia in *ob/ob* mice

After the 4-week feeding period, *ob/ob* mice fed the control diet had mild hyperlipidemia (Table 2). The serum triglyceride level was 1.3-fold higher and the serum cholesterol level was 2.7-fold higher in control-fed *ob/ob* mice than C57BL/6J mice. The Mukitake diet markedly lowered the triglyceride and cholesterol concentrations in the serum of *ob/ob* mice (Table 2). Additionally, Table 2 shows the serum lipoprotein profiles in C57BL/6J mice and *ob/ob* mice. Triglyceride levels in the chylomicron (CM) and very-low-density lipoprotein (VLDL) fractions tended to decrease (by 53% and 33%, respectively) and levels in the low-density lipoprotein (LDL) and high-density lipoprotein (HDL) fractions were decreased (by 45% and 34%, respectively) in the Mukitake group compared with the Control group in *ob/ob* mice. The cholesterol levels in all fractions were decreased in Mukitake-fed *ob/ob* mice. Moreover, the calculated atherogenic index (cholesterol ratio of [VLDL + LDL]/HDL) was significantly lower (by 41%) in Mukitake-fed *ob/ob* mice compared with control-fed *ob/ob* mice. These results suggest that dietary Mukitake can alleviate dyslipidemia in obese, diabetic *ob/ob* mice and has an anti-atherogenic property.

**Table 2:** **Effect of Mukitake diet on growth parameters in C57BL/6J and *ob/ob *mice**

	**Normal**	**Control**	**Mukitake**
	C57BL/6J	*ob/ob*	
Triglyceride (mg/dl)			
Total	23.7 ± 2.5 ^a^	54.4 ± 6.2 ^b^	33.2 ± 1.6 ^a^
CM	0.0283 ±0.0170	0.432 ± 0.227	0.205 ± 0.067
VLDL	13.2 ± 2.1	16.8 ± 3.7	11.3 ± 1.4
LDL	8.53 ± 0.45 ^a^	27.0 ± 1.7 ^b^	14.9 ± 0.7 ^c^
HDL	1.89 ± 0.06 ^a^	10.2 ± 1.3 ^b^	6.73 ± 0.46 ^c^
Cholesterol (mg/dl)			
Total	100 ± 5 ^a^	273 ± 6 ^b^	206 ± 7 ^c^
CM	0.0283 ± 0.0048 ^a^	0.252 ± 0.028 ^b^	0.157 ± 0.008 ^c^
VLDL	2.69 ± 0.34 ^a^	12.1 ± 1.5 ^b^	5.74 ± 0.35 ^c^
LDL	10.0 ± 1.0 ^a^	71.1 ± 2.7 ^b^	36.8 ± 1.9 ^c^
HDL	87.2 ± 4.2 ^a^	190 ± 4 ^b^	164 ± 5 ^c^
Atherogenic index			
(VLDL + LDL)/HDL	0.145 ± 0.010 ^a^	0.438 ±0.015 ^b^	0.259 ± 0.005 ^c^
Insulin (ng/ml)	0.886 ± 0.248 ^a^	52.1 ± 13.1 ^b^	56.3 ± 9.8 ^b^
Adiponectin (μg/ml)	29.6 ± 0.6 ^a^	11.6 ± 0.5 ^b^	12.3 ± 0.3 ^b^

Values are expressed as the mean **±** standard error for six mice.

^abc^ Different superscripted letters indicate a significant difference at *P* < 0.05.

CM; chylomicron, VLDL; very-low-density lipoprotein, LDL; low-density lipoprotein, HDL; high-density lipoprotein.

### Effect of dietary Mukitake on hyperinsulinemia and serum adiponectin levels in *ob/ob* mice

Insulin resistance, as well as compensatory hyperinsulinemia, are the essential first pathologic step in the development of NAFLD and dyslipidemia [25-27]. In fact, hepatic steatosis and hyperlipidemia have been proposed to be a feature of insulin resistance syndrome along with type 2 diabetes and visceral obesity [25-27]. Adiponectin is one of the most abundant secretory proteins produced by adipose tissue in rodents and humans and is suggested to play a protective role in insulin resistance [28]. In obese, diabetic *ob/ob* mice (Control group), serum insulin levels were markedly increased and serum adiponectin levels were significantly decreased compared with the C57BL/6J mice (Normal group) (Table 2). Our previous study indicated that powdered whole Mukitake and Mukitake extracts tended to alleviate hyperinsulinemia through increasing serum adiponectin levels in *db/db* mice [17,18]. In the present study, however, the Mukitake diet did not affect serum adiponectin levels or improve hyperinsulinemia in *ob/ob* mice. The results suggest that the alleviating effect of the Mukitake diet on the pathogenesis of NAFLD and dyslipidemia appears to be independent of the adiponectin level and hyperinsulinemia in *ob/ob* mice.

### Effect of dietary Mukitake on hepatic lipid metabolism in *ob/ob* mice

**Figure 3:**
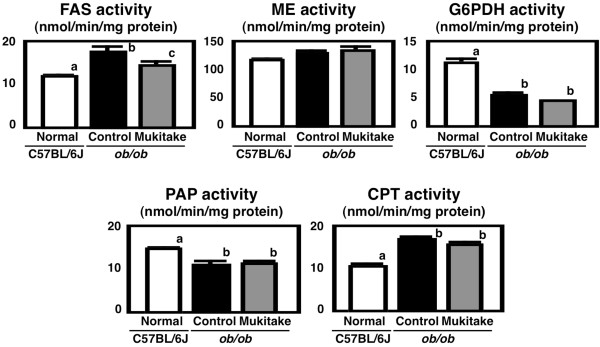
**Activities of hepatic enzymes related to triglyceride metabolism in C57BL/6J and *ob*/*ob *mice.** Mice were fed the control diet or Mukitake diet for 4 weeks. Values are expressed as the mean ± standard error for six mice. See Table 1 for the composition of diets. ^abc^ Different superscripted letters indicate a significant difference at *P* < 0.05.

The liver is the pivotal organ with regard to lipid metabolism; thus, its metabolic disruption induces lipid abnormality in the entire body. To further examine the effect of the Mukitake diet on the liver, the activities of hepatic enzymes related to triglyceride metabolism were analyzed (Figure 3). The activities of malic enzyme and G6PDH, enzymes related to lipogenesis, the activity of PAP, a key enzyme in triglyceride synthesis, and the activity of CPT, a key enzyme in fatty acid β-oxidation, were not altered by the Mukitake diet in *ob/ob* mice. On the other hand, the activity of FAS, a key enzyme in de novo fatty acid synthesis, was significantly elevated (by 50%) in control diet-fed *ob/ob* mice compared with the C57BL/6J mice and reduced (by 18%) in Mukitake-fed *ob/ob* mice compared with control diet-fed *ob/ob* mice. In agreement with the present results, previous reports showed that dietary intake of powdered whole Mukitake or both water extracts and ethanol-extracts from Mukitake prevented the development of NAFLD with the suppression of hepatic FAS activities in *db/db* mice [17,18]. Because differential analysis between Mukitake and Shiitake, mycelia from the same family, using liquid chromatography time-of –flight MS technology revealed that there are several compounds only exist in Mukitke extracts [18], further structural identification and evaluation of physiological properties (especially, suppressive effects on FAS activity) of the compounds would be necessary in future study. Additionally, given the fact that hepatic lipid abnormalities initiate a progression of liver damage and a sequence of lipoprotein changes [29,30], we suppose that the alleviation of NAFLD and dyslipidemia by the Mukitake diet was partially attributable to the suppression of de nove fatty acid synthesis in the liver.

## Conclusion

In conclusion, our present study showed that Mukitake supplementaion is beneficial for the inhibition of NAFLD development in leptin-deficient *ob/ob* mice besides in leptin-resistant *db/db* mice. Moreover, this is the first study showing that Mukitake has beneficial effects in attenuating possible atherosclerotic processes by reducing the atherogenic index in the serum.

## Abbreviations

ALT: Alanine aminotransferase; AST: Aspartate aminotransferase; CM: Chylomicron; CPT: Carnitine palmitoyltransferase; FAS: Fatty acid synthase; G6PDH: Glucose 6-phosphate dehydrogenase; HDL: High-density lipoprotein; LDL: Low-density lipoprotein; PAP: Phosphatidate phosphohydrolase; NAFLD: Non-alcoholic fatty liver disease; VLDL: Very-low-density lipoprotein.

## Competing interests

The authors declare that they have no competing interests.

## Authors’ contributions

NI and KN made substantial contributions to the conception and design of the study, performing the experiment, assembling, analyzing and interpreting the data and drafting the manuscript. MI and BS participated in the experimental work and in collecting, assembling, and analyzing the data. TY contributed to planning the experiment and discussing the results. All authors read and approved the final manuscript.

## References

[B1] Chang R Functional properties of edible mushrooms Nutr Rev 1996 54 S91 93 9110582 10.1111/j.1753-4887.1996.tb03825.x

[B2] Wasser SP Medical mushrooms as a source of antitumor and immunomodulating polysaccharides Appl Microbiol Biotechnol 2002 60 258 274 10.1007/s00253-002-1076-7 12436306

[B3] Ukawa Y Furuichi Y Kokean Y Nishii T Hisamatsu M Effect of hatakeshimeji (Lyophyllum decastes Sing.) mushroom on serum lipid levels in rats J Nutr Sci Vitaminol 2002 8 73 76 12026194 10.3177/jnsv.48.73

[B4] Zaidman B-Z Yassin M Mahajna J Wasser SP Medical mushroom modulators of molecular targets as cancer therpeutics Appl Microbiol Biotechnol 2005 67 453 468 10.1007/s00253-004-1787-z 15726350

[B5] Watanabe A Kobayashi M Hayashi S Kodama D Isoda K Kondoh M Kawase M Tamesada M Yagi K Protection against D-galactosamine-induced acute liver injury by oral administration of extracts from lentinus edodes mycelia Biol Pharm Bull 2006 29 1651 1654 10.1248/bpb.29.1651 16880621

[B6] Ukawa Y Izumi Y Ohbuchi T Takahashi T Ikemizu S Kojima Y Oral administration of the extract from Hatakeshimeji (Lyophyllum decastes Sing.) mushroom inhibits the development of atopic dermatitis-like skin lesions in NC/Nga mice J Nutr Sci Vitaminol 2007 53 293 196 10.3177/jnsv.53.293 17874836

[B7] Nagamori N Simple equipment cultivation of Panellus serotinus Kyushu J Forrest Res 2007 60 146 148

[B8] Kissebah AH Krakower GR Regional adiposity and morbidity Physiol Rev 1994 74 761 811 7938225 10.1152/physrev.1994.74.4.761

[B9] Formiguera X Canton A Obesity: epidemiology and clinical aspects Best Pract Res Clin Gastroenterol 2004 18 1125 1146 15561643 10.1016/j.bpg.2004.06.030

[B10] Fan JG Li F Cai XB Peng YD Ao QH Gao Y Effects of nonalcoholic fatty liver disease on the development of metabolic disorders J Gastroenterol Hepatol 2007 22 1086 1091 10.1111/j.1440-1746.2006.04781.x 17608855

[B11] Harrison SA Diehl AM Fat and the liver—a molecular overview Semin Gastrointest Dis 2002 13 3 16 11944631

[B12] Youssef W McCullough AJ Diabetes mellitus, obesity, and hepatic steatosis Semin Gastrointest Dis 2002 13 17 30 11944630

[B13] Nagao K Yanagita T Bioactive lipids in metabolic syndrome Prog Lipid Res 2008 47 127 146 10.1016/j.plipres.2007.12.002 18177744

[B14] Hummel KP Dickie MM Coleman DL Diabetes, a new mutation in the mouse Science 1966 153 1127 1128 10.1126/science.153.3740.1127 5918576

[B15] Zhang Y Proenca R Maffei M Barone M Leopold L Friedman JM Positional cloning of the mouse obese gene and its human homologue Nature 1994 372 425 432 10.1038/372425a0 7984236

[B16] Trak-Smayra V Paradis V Massart J Nasser S Jebara V Fromenty B Pathology of the liver in obese and diabetic ob/ob and db/db mice fed a standard or high-calorie diet Int J Exp Pathol 2011 92 413 421 10.1111/j.1365-2613.2011.00793.x 22118645 PMC3248077

[B17] Nagao K Inoue N Inafuku M Shirouchi B Morooka T Nomura S Nagamori N Yanagita T Mukitake mushroom (Panellus serotinus) alleviates nonalcoholic fatty liver disease through the suppression of monocyte chemoattractant protein-1 production in db/db mice J Nutr Biochem 2010 21 418 423 10.1016/j.jnutbio.2009.01.021 19423319

[B18] Inafuku M Nagao K Nomura S Shirouchi B Inoue N Nagamori N Nakayama H Toda T Yanagita T Protective effects of fractional extracts from Panellus serotinus on nonalcoholic fatty liver disease in obese, diabetic db/db mic Br J Nutr 2012 107 639 646 10.1017/S0007114511003485 21787451

[B19] American Institute of Nutrition Report of the American institute of nutrition ad hoc committee on standards for nutritional studies J Nutr 1977 107 1340 1348 874577 10.1093/jn/107.7.1340

[B20] Wang YM Nagao K Ujino Y Sakata K Higa K Inoue N Yanagita T Short-term feeding of conjugated linoleic acid does not induce hepatic steatosis in C57BL/6J mice J Nutr Sci Vitaminol 2005 51 440 444 10.3177/jnsv.51.440 16521704

[B21] Shirouchi B Nagao K Inoue N Furuya K Koga S Matsumoto H Yanagita T Dietary phosphatidylinositol prevents the development of nonalcoholic fatty liver disease in Zucker (fa/fa) rats J Agric Food Chem 2008 56 2375 2379 10.1021/jf703578d 18324772

[B22] Ikeda I Konno R Shimizu T Ide T Takahashi N Kawada T Nagao K Inoue N Yanagita T Hamada T Morinaga Y Tomoyori H Imaizumi K Suzuki K Campest-5-en-3-one, an oxidized derivative of campesterol, activates PPARα, promotes energy consumption and reduces visceral fat deposition in rats Biochim Biophys Acta 2006 1760 800 807 10.1016/j.bbagen.2006.02.017 16616424

[B23] Wang YM Nagao K Inoue N Ujino Y Shimada Y Nagao T Iwata T Kamegai T Yamauchi-Sato Y Yanagita T Isomer-specific anti-obese and hypolipidemic properties of conjugated linoleic acid in obese OLETF rats Biosci Biotechnol Biochem 2006 70 355 362 10.1271/bbb.70.355 16495650

[B24] Nagao K Yamano N Shirouchi B Inoue N Murakami S Sasaki T Yanagita T Effects of citrus auraptene (7-geranyloxycoumarin) on hepatic lipid metabolism in vitro and in vivo J Agric Food Chem 2010 58 9028 9032 10.1021/jf1020329 20681532

[B25] Marceau P Biron S Hould FS Marceau S Simard S Thung SN Kral JG Liver pathology and the metabolic syndrome X in severe obesity J Clin Endocrinol Metab 1999 84 1513 1517 10.1210/jc.84.5.1513 10323371

[B26] Marchesini G Brizi M Bianchi G Tomassetti S Bugianesi E Lenzi M McCullough AJ Natale S Forlani G Melchionda N Nonalcoholic fatty liver disease: a feature of the metabolic syndrome Diabetes 2001 50 1844 1850 10.2337/diabetes.50.8.1844 11473047

[B27] Marchesini G Bugianesi E Forlani G Cerrelli F Lenzi M Manini R Natale S Vanni E Villanova N Melchionda N Rizzetto M Nonalcoholic fatty liver, steatohepatitis, and the metabolic syndrome Hepatology 2003 37 917 923 10.1053/jhep.2003.50161 12668987

[B28] Matsuzawa Y Therapy Insight: adipocytokines in metabolic syndrome and related cardiovascular disease Nat Clin Pract Cardiovasc Med 2006 3 35 42 10.1038/ncpcardio0380 16391616

[B29] Day CP James OF Steatohepatitis: a tale of two “hits”? Gastroenterology 1998 114 842 845 10.1016/S0016-5085(98)70599-2 9547102

[B30] Matsuzawa Y Overproduction of very Low-density lipoproteins is the hallmark of the dyslipidemia in the metabolic syndrome Arterioscler Thromb Vasc Biol 2008 28 1225 1236 10.1161/ATVBAHA.107.160192 18565848

